# Genetic diversity, virulence genes, antimicrobial resistance, and biofilm formation of *Klebsiella pneumoniae* isolated from bovine mastitis milk in South Korea

**DOI:** 10.1128/spectrum.01343-25

**Published:** 2025-10-02

**Authors:** Hye Jeong Kang, Ju-Yeon You, Seung Hoe Kim, Jin-San Moon, Ha-Young Kim, Jae-Myung Kim, Hyun-Mi Kang

**Affiliations:** 1 Animal and Plant Quarantine Agency, Gimcheon, Republic of Korea; Universidad Maimonides, Buenos Aires, Argentina

**Keywords:** Antimicrobial resistance, biofilm, bovine mastitis, *Klebsiella pneumoniae*, multilocus sequence typing, virulence factors

## Abstract

*Klebsiella pneumoniae*, a zoonotic agent, is a causative pathogen of bovine mastitis. Despite its clinical relevance in dairy farms, studies on *K. pneumoniae* in bovine mastitis remain limited. Additionally, studies on *K. pneumoniae*’s genetic diversity and virulence characteristics in South Korea remain limited. Therefore, in this study, we aimed to elucidate the genetic diversity, antimicrobial resistance, virulence genes, and biofilm-forming capacity of 29 *K*. *pneumoniae* strains isolated from bovine mastitis milk samples in South Korea between 2017 and 2023. Multilocus sequence typing revealed 23 sequence types, four of which were novel, indicating substantial genetic heterogeneity and the absence of a dominant clonal lineage. Excluding intrinsic resistance, the highest resistance rates were observed for tetracycline (34.5%) and sulfisoxazole (31.0%), whereas resistance to the other antibiotics tested ranged from 0% to 20.7%. In addition, multidrug resistance (MDR) was noted in 20.7% of isolates. Virulence gene analysis revealed that most isolates carried the *ureA*, *uge*, *wabG*, and *fimH* genes, whereas *allS*, *rmpA*, *iucB*, and *iroNB* were not detected. Two isolates exhibited a hypermucoviscous phenotype, and one belonged to the capsular serotype K2. All isolates demonstrated biofilm-forming ability, with moderate-to-strong production observed in over 89.0% of cases, indicating potential for persistence and treatment challenges. In conclusion, *K. pneumoniae* isolates from mastitis milk carried multiple virulence genes and showed MDR as well as robust biofilm formation. Therefore, continued surveillance and further characterization of *K. pneumoniae* are needed to support mastitis control and protect public health.

**IMPORTANCE:**

*Klebsiella pneumoniae* is an emerging environmental pathogen associated with clinical mastitis in dairy cows, raising concerns regarding antimicrobial resistance and public health. To the best of our knowledge, this study provides the first comprehensive characterization of *K. pneumoniae* isolates from mastitis milk in South Korea, including analyses of genetic diversity, antimicrobial resistance, virulence factors, and biofilm formation. The findings advance our current understanding of *K. pneumoniae* associated with bovine mastitis and highlight the need for continued surveillance that will contribute to mastitis control efforts and safeguard public health.

## INTRODUCTION

Bovine mastitis is one of the most economically significant diseases in the dairy industry, leading to reduced milk production and increased veterinary costs (1). Based on clinical signs, mastitis is classified into clinical and subclinical types (1). Although approximately 150 bacterial species are suspected to be linked to mastitis, most cases are caused by a relatively small number of bacterial groups, predominantly by 10 key taxa (2). Mastitis-causing bacteria are classified as either contagious or environmental pathogens, depending on their transmission routes (1–3). While contagious mastitis pathogens spread among cows, environmental pathogens primarily originate from the dairy cow’s surroundings (1, 3). The most significant environmental mastitis pathogens include *Streptococcus uberis*, *Escherichia coli*, and *Klebsiella pneumoniae* (1, 3).

*Klebsiella pneumoniae*, a Gram-negative encapsulated bacterium, is widely recognized as a major zoonotic pathogen. In humans, *K. pneumoniae* is responsible for pneumonia, urinary tract infections, bacteremia, and liver abscesses (4). It is broadly categorized into classical (cKp), hypervirulent (hvKp), and multidrug-resistant (MDR) types, with cKp typically linked to hospital-acquired infections in immunocompromised individuals, while hvKp is capable of causing invasive disease even in healthy hosts (4–6). *K. pneumoniae* is recognized as one of the leading causes of clinical mastitis in dairy cows and is often characterized by a prolonged course with mild-to-moderate symptoms and significant milk production losses, although it can progress to severe forms, resulting in an increased risk of mortality or culling (1, 7–9). Over the last decade, *K. pneumoniae* has been more frequently isolated from bovine milk samples globally, and it is now considered a major environmental mastitis pathogen commonly found in water, soil, vegetation, and on animal mucosal surfaces (1, 2, 10). Cases of mastitis caused by hvKp and MDR *K. pneumoniae* have been increasingly reported, highlighting emerging concerns regarding its pathogenic potential in dairy cows (9–12).

Biofilm formation is a key virulence mechanism of *K. pneumoniae*, facilitating its survival in hostile environments by conferring protection against host immune defenses and antimicrobial agents (3, 4). It has been estimated that between 65% and 80% of bacterial infections involve the production of biofilms, and human clinical *K. pneumoniae* isolates are frequently characterized by this trait (4, 13). The development of these biofilms is mediated by the coordinated expression of certain virulence-associated genes, among which *fimH* promotes adhesion to epithelial surfaces during early colonization, whereas *rmpA* and *wcaG* regulate capsular polysaccharide synthesis and contribute to the structural integrity of the biofilm matrix (4). In addition, siderophore-related genes, such as *kfuBC* and *ybtA*, enhance bacterial adaptation under iron-limited conditions (4). In the context of bovine mastitis, by promoting adherence to host tissues and enabling the evasion of immune responses, thereby potentially compromising the efficacy of antimicrobial treatment, biofilm-forming *K. pneumoniae* strains may facilitate persistent intramammary infections (3, 14).

Molecular epidemiological approaches enhance our understanding of host adaptation and pathogenesis by revealing genetic factors involved in pathogen evolution, interspecies gene transfer, and potential targets for intervention (15). Tools, such as multilocus sequence typing (MLST), virulence gene profiling, and whole-genome sequencing, have been employed to investigate the adaptation of major pathogens to bovine hosts and mammary glands, supporting their application in outbreak investigations and on-farm disease management (15, 16).

Despite its clinical relevance in dairy farms, studies on *K. pneumoniae* in bovine mastitis have been limited (9, 10, 12). In South Korea, previous studies on *K. pneumoniae*, as the causative agent of mastitis, have primarily focused on its antimicrobial susceptibility, with limited investigation into its genetic diversity and virulence characteristics (17, 18). Therefore, this study aimed to characterize the genetic diversity, antimicrobial susceptibility, virulence-associated genes, and biofilm-forming abilities of *K. pneumoniae* isolates from bovine mastitis milk in South Korea.

## RESULTS

### Sequence types of *K. pneumoniae*

**Fig 1:**
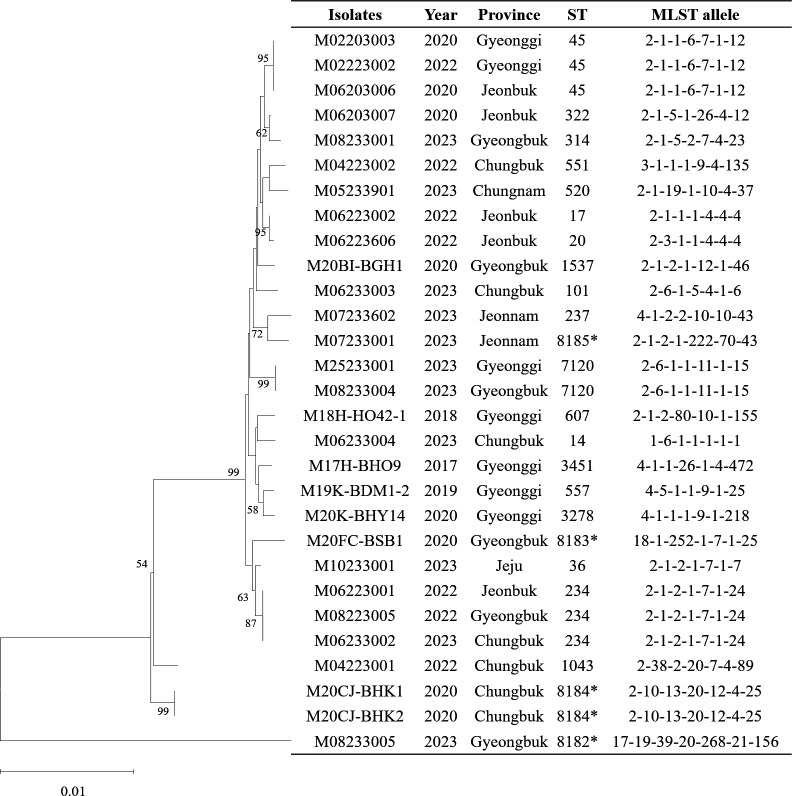
MLST-based phylogenetic tree of *Klebsiella pneumoniae* isolated from bovine mastitis milk. A phylogenetic tree of the 29 *Klebsiella pneumoniae* isolates was constructed using MLST sequences and the neighbor-joining method with the Jukes-Cantor model. Bootstrap analysis with 1,000 replicates was performed. The isolate names, collection years, and geographic origins were also included. NT, non-typeable. *These isolates represent the novel STs identified in this study.

MLST was performed to analyze the genetic classification of the isolates within species. Among the 29 *K*. *pneumoniae* isolates studied, 23 distinct sequence types were identified, including four novel types (Fig. 1). Among the identified sequence types, ST45 and ST234 were each detected in three isolates (10.3%), followed by ST7120 and ST8184 in two isolates (6.9%). The remaining 19 sequence types (ST14, ST17, ST20, ST36, ST101, ST237, ST314, ST322, ST520, ST551, ST557, ST607, ST1043, ST1537, ST3278, ST3451, ST8182, ST8183, and ST8185) were each represented by a single isolate (3.4%). Of them, ST8182, ST8183, ST8184, and ST8185 were identified for the first time.

**Fig 2:**
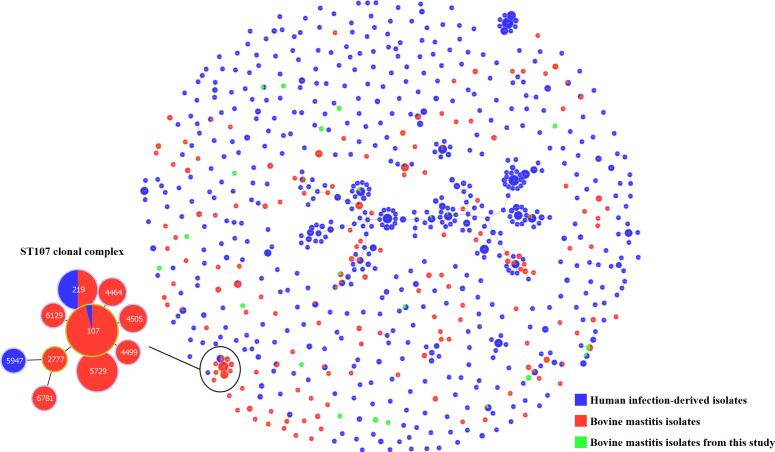
Minimum spanning tree analysis of *Klebsiella pneumoniae* isolates from human infection-derived and bovine mastitis milk based on MLST allelic profiles. A minimum spanning tree of *K. pneumoniae* isolates was generated using the geoBURST algorithm in PhyloViz 2.0, based on multilocus sequence typing allelic profiles and the single-locus variant criterion. Each node represents a unique sequence type (ST), and the node size is proportional to the number of isolates sharing the same ST. Colors indicate the origin of isolates: blue represents human infection-derived isolates, red represents bovine mastitis isolates, and green represents bovine mastitis isolates. Edges connect STs that differ in only one of the seven housekeeping loci.

To investigate the genetic relationship between *K. pneumoniae* isolates from human infections and bovine mastitis, MLST data of globally reported strains were retrieved from the Pasteur MLST database and analyzed using minimum spanning tree (MST) (Fig. 2). Most bovine mastitis isolates (red and green) were either dispersed among human infection-derived isolates (blue) or appeared as singletons in the MST.

### Antimicrobial susceptibility of *K. pneumoniae*

**Fig 3:**
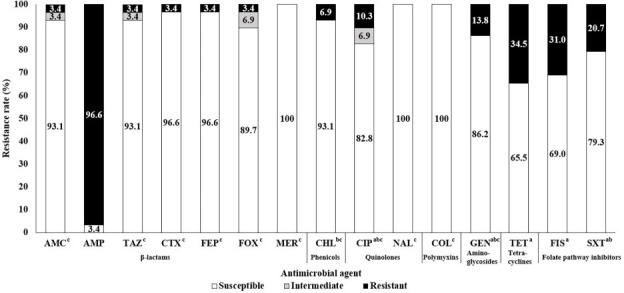
Antimicrobial susceptibility of *Klebsiella pneumoniae* isolated from bovine mastitis milk. Superscript letters (a–c) following the antimicrobial names on the *x*-axis indicate statistically significant differences in antibiotic resistance rates (*P* < 0.05). Ampicillin resistance was excluded from statistical analysis because of the intrinsic resistance of *K. pneumoniae* to this antimicrobial. AMC, amoxicillin/clavulanic acid; AMP, ampicillin; TAZ, ceftazidime; CTX, cefotaxime; FEP, cefepime; FOX, cefoxitin; MER, meropenem; CHL, chloramphenicol; CIP, ciprofloxacin; NAL, nalidixic acid; COL, colistin; GEN, gentamicin; TET, tetracycline; FIS, sulfisoxazole; SXT, trimethoprim/sulfamethoxazole.

The antimicrobial susceptibility of *K. pneumoniae* isolates was assessed by determining their MICs (Fig. 3). The highest resistance rate was observed for ampicillin (96.6%), which was expected due to the intrinsic resistance of *K. pneumoniae* to this antimicrobial agent. Among the remaining antibiotics, the highest resistance rates were observed for tetracycline (34.5%) and sulfisoxazole (31.0%), followed by trimethoprim/sulfamethoxazole (20.7%). Resistance to other antimicrobials was relatively low, with only 3.4% of the isolates being resistant to amoxicillin/clavulanic acid, cefepime, cefotaxime, cefoxitin, and ceftazidime. Meanwhile, all isolates were susceptible to colistin, meropenem, and nalidixic acid. Statistically significant differences in the resistance rates were observed among antibiotics (*P* < 0.05). Additionally, six isolates (20.7%) were classified as MDR, including M02203003, M05233901, M06233003, M25233001, M08233004, and M19K-BDM1-2, based on resistance to at least three antibiotic classes, excluding intrinsic resistance.

### Antimicrobial resistance genes of *K. pneumoniae*

A total of 18 antimicrobial resistance (AMR) genes covering six antibiotic classes were detected in *K. pneumoniae* isolates using PCR (Table 1). All isolates harbored at least one AMR gene. Among the tested genes, *bla_SHV_* was the most prevalent, found in 93.1% of the isolates. Aminoglycoside resistance genes (*strA* and *strB*) were detected in 31.0% of the isolates, whereas sulfonamide resistance genes (*sulI* and *sulII*) were identified in 20.7–24.1%. Tetracycline resistance genes (*tetA*, *tetB*, and *tetC*) were present in 13.8–20.7% of isolates, followed by β-lactam resistance genes (*bla_OXA_*, *bla_TEM_*, and *bla_CTX-M_*) in 3.4–10.3%, trimethoprim resistance gene (*dfrXII*) in 10.3%, and phenicol resistance gene (*floR*) in 3.4%. In contrast, genes *ampC*, *bla_IMP_*, *aph(3')-lia*, *dhfrV*, and *sulIII* were not detected in any of the isolates.

**TABLE 1:** Antimicrobial resistance patterns and the occurrence of resistance genes in *Klebsiella pneumoniae* isolated from bovine mastitis milk^*b*^

Antimicrobial resistance	*N*	Resistance genes																	
		β-lactams					Phenicols	Aminoglycosides			Trimethoprim			Sulfonamides			Tetracyclines		
		*ampC*	*blaTEM*	*blaSHV*	*blaOXA*	*blaCTX-M*	*floR*	*strA*	*strB*	*aph(3')-lia*	*blaIMP*	*dhfrV*	*dhfrXIII*	*sulI*	*sulII*	*sulIII*	*tetA*	*tetB*	*tetC*
AMP	16			16	1		1								1			1	3
AMP/TET	3			3				2	2								1	1	
AMP/FIS	2			2				2	2						2				2
Pan-susceptible	1							1	1										
AMP/FIS/TET/SXT	1			1				1	1				1	1	1		1		
AMP/GEN/ FIS/ TET^*a*^	1		1	1				1	1						1			1	
AMP/CHL/FIS/TET/SXT^*a*^	1			1										1			1		
AMP/CIP/FIS/TET/SXT^*a*^	1			1									1	1			1		
AMP/CHL/GEN/FIS/TET/SXT^*a*^	1			1										1					
AMC/AMP/FOX/CIP/GEN/FIS/TET/SXT^*a*^	1				1			1	1					1	1		1	1	1
AMP/FEP/CTX/CAZ/CIP/GEN/FIS/TET/SXT^*a*^	1			1	1	1		1	1				1	1	1		1		
Distribution of resistance genes		0	3.4	93.1	10.3	3.4	3.4	31.0	31.0	0	0	0	10.3	20.7	24.1	0	20.7	13.8	20.7

^*a*^ Multidrug resistance.

^*b*^ *N*, the number of isolates exhibiting antimicrobial resistance phenotype patterns; AMC, amoxicillin/clavulanic acid; AMP, ampicillin; FEP, cefepime; CTX, cefotaxime; FOX, cefoxitin; CAZ, ceftazidime; CHL, chloramphenicol; CIP, ciprofloxacin; GEN, gentamicin; FIS, sulfisoxazole; TET, tetracycline; SXT, trimethoprim/sulfamethoxazole.

### Capsular serotypes and the hypermucoviscous phenotype of *K. pneumoniae*

PCR was used to examine the distribution of the most common capsular serotypes associated with hypervirulent *Klebsiella pneumoniae*, specifically K1, K2, K5, K20, K54, and K57 (Table 2). Of the 29 isolates, only one (3.4%) was identified as serotype K2, whereas serotypes K1, K5, K20, K54, and K57 were not detected. The remaining 28 isolates were predicted to belong to serotypes not tested in this study.

**TABLE 2:** Summary of biofilm formation, capsular types, string test, and virulence gene profiles in *Klebsiella pneumoniae* isolated from bovine mastitis milk

Isolates	Biofilm	Capsular type	String test	Virulence genes										
				*allS*	*rmpA*	*wcaG*	*ureA*	*uge*	*wabG*	*fimH*	*kfuBC*	*ybtA*	*iucB*	*iroNB*
M17H-BHO9	Weak						+		+	+				
M18H-HO42-1	Moderate						+	+	+	+				
M19K-BDM1-2	Strong						+	+	+	+				
M06203006	Moderate						+	+	+	+				
M06203007	Moderate						+		+	+				
M02203003	Strong						+	+	+	+		+		
M20K-BHY14	Strong						+	+	+	+				
M20N-BHK2	Moderate						+	+	+	+				
M20BI-BGH1	Strong						+	+	+	+				
M20CJ-BHK1	Moderate		+^*a*^				+	+	+	+				
M20CJ-BHK2	Moderate		+				+	+	+	+				
M20FC-BSB1	Moderate						+	+	+	+				
M20GD-BHK3	Strong						+	+	+	+				
M20GD-BHK4	Moderate						+	+	+	+				
M02223002	Moderate						+	+	+	+		+		
M04223001	Moderate						+	+	+	+				
M04223002	Moderate						+	+	+	+				
M06223001	Moderate						+	+	+	+				
M06223002	Weak						+	+	+	+				
M06223606	Weak						+	+	+	+				
M08223005	Strong						+	+	+	+				
M08233001	Strong						+	+	+	+				
M25233001	Moderate						+	+	+	+	+			
M05233901	Strong	K2					+	+	+	+				
M06233002	Moderate						+	+	+	+				
M06233003	Moderate						+	+	+	+	+			
M06233004	Moderate					+	+	+	+	+	+			
M07233001	Strong						+	+	+	+				
M07233602	Strong						+	+	+	+				
M08233004	Moderate						+	+	+	+	+			
M08233005	Strong						+	+	+	+	+			
M10233001	Strong						+	+	+	+				
Distribution (%)			6.9	0	0	3.4	100	93.1	100	100	17.2	6.9	0	0

^*a*^ "+” denotes a positive string test or PCR detection of the corresponding virulence gene.

The hypermucoid characteristics of *K. pneumoniae* were confirmed using a string test (Table 2). Among the 29 isolates, two (6.9%) exhibited a hypermucoviscous phenotype.

### Virulence genes of *K. pneumoniae*

Virulence-associated genes commonly implicated in *K. pneumoniae* pathogenicity were detected via PCR (Table 2). All isolates harbored the *wabG*, *ureA*, and *fimH* genes, while *uge* was detected in 27 (93.1%), *kfuBC* in 5 (17.2%), *ybtA* in 2 (6.9%), and *wcaG* in 1 isolate (3.4%). *allS*, *rmpA*, *iroNB*, and *iucB* were not detected in any of the isolates. The most frequently observed virulence gene profile was *ureA* + uge *+ wabG + fimH*, identified in 20 isolates (69.0%).

### Biofilm formation of *K. pneumoniae*

The biofilm formation ability of *K. pneumoniae* isolates was assessed using a microtiter plate assay (Table 2). All isolates demonstrated biofilm-forming capacity, with moderate biofilm formation observed in 15 isolates (51.7%), representing the highest proportion. Strong biofilm formation was observed in 11 isolates (37.9%), while weak biofilm formation was noted in three isolates (10.3%).

## DISCUSSION

The implementation of mastitis control programs has significantly reduced the prevalence of contagious causative pathogens but has concurrently led to an increased incidence of opportunistic Gram-negative bacteria, particularly *K. pneumoniae* (7, 19, 20). Despite being well-characterized as an opportunistic pathogen in humans, the characteristics of *K. pneumoniae* in bovine mastitis remain understudied. In this study, we analyzed the genetic diversity, antimicrobial susceptibility, virulence factors, and biofilm-forming abilities of *K. pneumoniae* isolates from bovine mastitis milk in South Korea, intending to obtain a better understanding of their pathogenic and epidemiological characteristics.

MLST-based typing has been extensively used in *K. pneumoniae* research to identify specific clones, and the classification of sequence types holds clinical significance (6, 21). In our study, MLST analysis identified 23 distinct sequence types, including four novel types, among a total of 29 *K*. *pneumoniae* isolates. Our findings highlight the high level of genetic diversity of *K. pneumoniae* isolates from bovine mastitis in South Korea and confirm the absence of a predominant epidemic clone, which is consistent with the findings of previous studies reporting high genetic diversity among *K. pneumoniae* isolates from bovine mastitis milk in the USA, Scotland, and China (11, 21, 22). This marked heterogeneity suggests that *K. pneumoniae* infections may arise from multiple environmental sources, such as feces, alleyways, bedding, water, and milking equipment, rather than clonal dissemination within farms. These findings support its classification as an environmental mastitis pathogen and underscore the importance of environmental hygiene and farm-level management in preventing transmission (3, 8, 19).

MLST analysis showed that while most bovine mastitis isolates were dispersed among human infection-derived isolates or appeared as singletons, a notable exception was the ST107 clonal group, which predominantly consisted of bovine isolates and formed a distinct clonal cluster. This observed grouping may indicate a potential host-associated lineage within *K. pneumoniae*. However, as the MLST data set used in this analysis comprised isolates from only six countries, this clustering may be influenced by sampling bias. Further investigation with a more comprehensive and geographically diverse data set is therefore warranted.

Although the increasing prevalence of antibiotic-resistant pathogens poses a significant global challenge, the complexity of bacteria responsible for intramammary infections in dairy cows makes antibiotic therapy the primary approach for managing bovine mastitis (10, 23). Comparison to a previous study conducted by our team between 2003 and 2008 revealed a reduction in the resistance to some antimicrobials. However, caution is warranted when comparing resistance data across studies due to differences in sample sources, timing of collection, testing methods, and interpretation criteria (24). The previous study reported resistance rates of 21.8% for chloramphenicol and gentamicin as well as 45.5% for tetracycline (17). In contrast, herein, we found lower rates of 6.9%, 13.8%, and 34.5%, respectively, suggesting a downward trend. This observed reduction may reflect the cumulative effects of improved mastitis control practices, including more judicious antimicrobial use, better milking hygiene, standardized treatment protocols, and routine bacterial monitoring (23). In South Korea, national mastitis control programs are coordinated by regional livestock health centers and diagnostic laboratories. These programs conduct annual surveillance, including pathogen identification and antimicrobial susceptibility testing, sharing the results with veterinarians and farmers to support evidence-based treatment and prevention. While these ongoing efforts may have contributed to the reduced resistance observed, further research is needed to determine their direct impact and identify specific contributing factors.

ESBL enzymes are capable of hydrolyzing penicillins, extended-spectrum cephalosporins, and the monobactam aztreonam, which limits treatment options (16, 25). ESBL-producing *K. pneumoniae* strains have been increasingly reported in both human and veterinary settings, raising concerns about antimicrobial resistance and potential zoonotic transmission (26, 27). In this study, a single ESBL-producing isolate was identified from a bovine mastitis case. Although this isolated finding does not suggest widespread dissemination among dairy farms, it warrants close attention due to its clinical relevance. Given this enzymatic activity, along with the increasing prevalence of ESBL producers in both humans and animals, the presence of an ESBL-producing strain in bovine milk highlights the importance of continued surveillance and prudent antimicrobial use to mitigate future risks to animal and public health.

The hypermucoviscous phenotype of *K. pneumoniae* is widely associated with invasive infections in healthy individuals, generally leading to the classification of this species as “hypervirulent” (5, 6). In the present study, we identified two isolates with a hypermucoviscous phenotype, representing a distribution rate of 6.9%, which is lower than the 14.5% reported for clinical mastitis cases in China (11). In human clinical isolates obtained in Spain, 13.39% of *K. pneumoniae* strains were found to be characterized by a hypermucoviscous phenotype (28). Notably, we failed to detect any major hypervirulence-associated genes, such as *rmpA*, *ybtA*, and *iucB*, in our hypermucoviscous isolates, which is consistent with the finding that neither *magA* nor *rmpA* was detected in hypermucoviscous *K. pneumoniae* strains isolated from clinical mastitis cases in China (11). Similar observations have been reported in human clinical strains, where hypermucoviscosity was not invariably correlated with the presence of these genes, and vice versa, with one study reporting that only 41.18% of hypermucoviscous strains carried both *rmpA* and *iucA* (28). These findings would thus tend to indicate that hypermucoviscosity and virulence may be influenced by additional regulatory mechanisms, which could account for the absence of any specific virulence determinants in phenotypically virulent strains (28).

The polysaccharide capsule is a major virulence factor in *K. pneumoniae* that protects it from phagocytosis, complement activation, antimicrobial peptides, and antibody-mediated opsonization, thereby enabling immune evasion (5, 6). Among capsular serotypes, K1, K2, K5, K20, K54, and K57 are the most frequently detected and highly virulent serotypes in human infections (5, 6). In our study, a single isolate (3.4%) was identified as the highly virulent serotype K2. Consistently, a study conducted in the USA reported a low prevalence (0–2.2%) of these serotypes, whereas a study from China found a higher occurrence of K57 (45.0%) (11). The low frequency of highly virulent serotypes commonly associated with human infections among our isolates was consistent with their virulence gene profiles. While all our isolates carried *wabG*, *ureA*, and *fimH*, which are commonly associated with adhesion, urease activity, and capsular biosynthesis, respectively, major hypervirulence-associated genes linked to invasive human infections, such as *rmpA*, *iroNB*, *iucB*, and *allS*, were not detected (5–7). Consistent with our findings, key virulence determinants encoding acquired siderophores and hypermucoidy have been infrequently identified in *K. pneumoniae* isolates recovered from patients with Scottish bovine mastitis (22). In contrast, Kamelia et al. reported that *magA*, *uge*, *kfu*, *rmpA*, and aerobactin are key virulence genes in invasive mastitis-causing *K. pneumoniae* strains in Egypt (12). In addition, in the present study, Yersiniabactin, a siderophore system strongly associated with pathogenicity and invasive infections, was detected in two (6.9%) of the assessed bovine mastitis isolates, a prevalence that is markedly lower than that reported for human clinical isolates from Spain (40.9% for *ybtS*) (28). In human hospital-associated ST101 isolates, yersiniabactin has been reported in 87% of cases, but it was not detected in strains of livestock, food, or healthy community origin (29). These differences may reflect host-specific selective pressures, ecological niche adaptation, or the absence of a fitness advantage for certain virulence factors in the bovine mammary gland environment (29). Taken together, our findings suggest that *K. pneumoniae* isolates associated with bovine mastitis in South Korea may exhibit a distinct virulence profile compared to the hypervirulent strains commonly found in human infections. Prevention of the emergence and spread of hypervirulent *K. pneumoniae* requires continued surveillance and further studies to clarify its pathogenicity in dairy cows and zoonotic potential.

Biofilm formation protects *K. pneumoniae* from antimicrobial agents and host immune responses, thereby promoting immune evasion and persistent intramammary infections through enhanced colonization and survival in the udder environment (3, 7). In our study, all the isolates exhibited biofilm formation, with most isolates showing either moderate (51.7%) or strong (37.9%) biofilm production. Clinical mastitis isolates from China have been reported to show a lower prevalence of biofilm formation (77.1%), among which 35.1%, 23.7%, and 18.3% of strains were classified as weak, moderate, and strong biofilm formers, respectively (11), whereas a higher prevalence of biofilm formation has been reported for human-derived clinical isolates, among which 54%, 29%, and 14% of strains were classified as strong, moderate, and weak biofilm formers, respectively, and only 2.8% identified as non-biofilm formers (30). These findings indicate that *K. pneumoniae* isolates from bovine mastitis possess biofilm-forming abilities that may contribute to their persistence in the udder environment. Additionally, our findings revealed that *fimH*, a gene associated with fimbrial adhesion, was universally detected in all isolates, whereas *wcaG*, which has been linked to biofilm formation in human bloodstream infections, was present at a low frequency (31–33). Consistent with our findings, Wusiman et al. reported that most *K. pneumoniae* isolates from bovine mastitis formed biofilms with a high *fimH* and low *wcaG* prevalence (11). Given that *fimH* is involved in fimbrial adhesion during early biofilm formation (31, 32), its predominant detection among bovine isolates suggests a potential role in the establishment of intramammary infection, which should be further investigated to clarify its functional significance. Furthermore, as our biofilm assays were performed *in vitro*, additional studies are needed to understand how these findings relate to biofilm behavior and pathogenicity in the mammary gland *in vivo*.

Given that our analysis was limited to isolates obtained from bovine mastitis milk samples, caution should be exercised when seeking to generalize these findings to *K. pneumoniae* strains isolated from other sources. Accordingly, further comparative studies involving human or environmental isolates are warranted to gain broader epidemiological insights.

In conclusion, our study showed that *K. pneumoniae* isolates from bovine mastitis milk in South Korea are genetically diverse, and no epidemic clones were identified. Notably, the identification of MDR isolates raises concerns about antimicrobial efficacy and potential public health implications. Of particular importance is the detection of the hypervirulence-associated serotype *K. pneumoniae* in mastitis milk samples. Moreover, all the isolates demonstrated biofilm-forming abilities, which may help them adhere to mammary tissue and persist in the udder. The current findings suggest that continuous monitoring and characterization of *K. pneumoniae* in mastitis milk are necessary to advance control strategies and protect public health.

## MATERIALS AND METHODS

### Isolation and identification of *K. pneumoniae*

As part of the National Bovine Mastitis Prevention System, 29 *K*. *pneumoniae* isolates were obtained from 20 regional laboratories and centers in South Korea (Table S1). These isolates were derived from the quarter milk of lactating cows suspected of having clinical or subclinical mastitis between 2017 and 2023. Bacteria were isolated according to the National Mastitis Council guidelines (34). The isolates were sent to the Animal and Plant Quarantine Agency for further characterization. Species identification was conducted using matrix-assisted laser desorption ionization-time-of-flight mass spectrometry (MALDI-TOF MS; bioMérieux, Marcy L’Etoile, France) in accordance with the manufacturer’s protocol. All isolates were stored at −80 ℃.

### Multilocus sequence typing and phylogenetics

MLST of *K. pneumoniae* isolates was performed based on a previously described method (35) using seven housekeeping genes (*gapA*, *infB*, *mdh*, *pgi*, *phoE*, *rpoB*, and *tonB*). Allele assignment and sequence type (ST) identification were performed using the *Klebsiella* MLST database (https://bigsdb.pasteur.fr/cgi-bin/bigsdb/bigsdb.pl?db=pubmlst_klebsiella_seqdef). A phylogenetic tree was constructed based on the MLST data using MEGA10 software. The tree was generated using the neighbor-joining method with the Jukes-Cantor model, and bootstrap analysis was performed with 1,000 replicates to ensure reliability.

MLST data were used to analyze the genetic relationships between *K. pneumoniae* isolates from human infections and bovine mastitis. Both human infection-derived and bovine milk isolates were obtained from the Pasteur MLST database (https://bigsdb.pasteur.fr/klebsiella/) by filtering for isolates with a documented infection source and a corresponding host (Table S2). Additionally, the bovine mastitis isolates obtained in this study were included for comparative analysis. MST was constructed using the goeBURST algorithm in PHYLOViZ 2.0. The tree was generated at the single-locus variant (SLV) level, which connects sequence types (STs) that differ at only one of the seven housekeeping loci. Each node represents a unique ST, with size proportional to the number of isolates. The isolates were color-coded by origin as follows: blue for human infection-derived isolates (*n* = 1,297), red for previously reported bovine mastitis isolates (*n* = 367), and green for bovine mastitis isolates newly obtained in this study (*n* = 29).

### Antimicrobial susceptibility testing

The antimicrobial susceptibility of *K. pneumoniae* isolates was evaluated using the broth microdilution method, in accordance with the CLSI guideline M100 (36). The minimum inhibitory concentration (MIC) was measured using a Sensitire panel KRNV6F (Trek Diagnostics, Cleveland, OH, USA), following the manufacturer’s protocol. The Sensitire panel KRNV6F included sixteen antimicrobials at various concentrations (μg/mL): amoxicillin/clavulanic acid (2/1–32/16), ampicillin (2–64), cefepime (0.25-16), cefotaxime (0.5–8), cefoxitin (1–32), ceftazidime (1–16), chloramphenicol (2–64), ciprofloxacin (0.12–16), colistin (2–16), gentamicin (1–64), meropenem (0.25-4), nalidixic acid (2–128), streptomycin (16–128), sulfisoxazole (16–256), tetracycline (2–128), and trimethoprim/sulfamethoxazole (0.12/2.38–4/76). The MIC values were interpreted based on the CLSI (2020) (36). *Escherichia coli* ATCC 25922 was used as the quality control strain.

### Detection of capsular serotypes, virulence genes, and antimicrobial resistance genes

Genomic DNA was extracted from *K. pneumoniae* isolates using the Maxwell RSC PureFood GMO & Authentication Kit (Promega, USA), following the manufacturer’s protocol adapted for bacterial samples. Detection of capsular serotypes (K1, K2, K5, K54, K57, K20), virulence genes, including those related to allantoin metabolism (*allS*), capsular polysaccharides (*wcaG*, *rmpA*), lipopolysaccharides (*wabG*, *ureA*, *uge*), pili (*fimH*), siderophores (*kfuBC*, *iroNB*, *ybtA*, *iucB*), and antimicrobial resistance genes, including those related to aminoglycosides (*strA*, *strB*, *aph(3')-lia*), β-lactams (*ampC*, *bla_TEM_*, *bla_SHV_*, *bla_OXA_*, *bla_CTX-M_*, *bla_IMP_*), phenicols (*floR*), sulfonamides (*sulI*, *sulII*, *sulIII*), tetracyclines (*tetA*, *tetB*, *tetC*), and trimethoprim (*dhfrV*, *dfrXIII*), was performed in *K. pneumoniae* isolates using PCR as previously described (31, 37–43). Details of the primers and amplification conditions are provided in Table S3.

### String test

A string test was conducted to evaluate the hypermucoviscosity of *K. pneumoniae* isolates, as previously described (5). Briefly, isolates were streaked onto blood agar plates (BAP) and incubated at 37℃ overnight. Colonies were stretched using a loop on the agar surface, and those producing viscous filaments of 5 mm or longer were classified as hypermucoviscous.

### Biofilm assay

The biofilm formation ability of *K. pneumoniae* isolates was evaluated using a microtiter plate assay, as described in previous studies (44). Briefly, a single colony was inoculated in 5 mL of BHI broth and incubated at 35℃ for 24 h. The overnight culture was diluted at a ratio of 1:125 into fresh 5 mL BHI broth, and 200 µL of the diluted culture was added to each well of a 96-well plate. The plates were incubated at 35℃ for 24 h. After incubation, the wells were washed three times with distilled water (DW). The biofilm was stained with 200 µL of 0.1% crystal violet at room temperature for 15 min. The plate was then washed thrice with DW and allowed to dry. The stained biofilm was dissolved with 200 µL of 70% ethanol for 15 min. Finally, 100 µL of the dissolved solution was transferred to a new 96-well plate, and the absorbance was measured at 570 nm. The isolates were categorized based on their optical density (OD) readings as follows: non-adherent (OD ≤ OD of the negative control), weak (OD of the negative control < OD ≤ 2 × OD of the negative control), moderate (2× OD of the negative control < OD ≤ 4× OD of the negative control), or strong (OD > 4× OD of the negative control) (45).

### Statistical analysis

Statistical analysis was performed using the Pearson’s chi-squared or Fisher’s exact test with Bonferroni correction to assess the differences in antimicrobial resistance rates among antibiotics. Statistical significance was set at *P* < 0.05. All analyses were conducted using SPSS Statistics software, version 25 (IBM Corp., Armonk, NY, USA).

## SUPPLEMENTAL MATERIAL

Table S1 spectrum.01343-25-s0001.xlsx Characteristics of *Klebsiella pneumoniae* isolates from bovine mastitis milk in South Korea.

Table S2 spectrum.01343-25-s0002.xlsx Information on *Klebsiella pneumoniae* isolates from human infections and bovine mastitis used for MLST-based comparative analysis from the Pasteur MLST database

Table S3 spectrum.01343-25-s0003.xlsx The primers and amplification conditions used in this study.

ASM does not own the copyrights to Supplemental Material that may be linked to, or accessed through, an article. The authors have granted ASM a non-exclusive, world-wide license to publish the Supplemental Material files. Please contact the corresponding author directly for reuse.
